# Silica Nanoparticles Induce Oxidative Stress and Autophagy but Not Apoptosis in the MRC-5 Cell Line

**DOI:** 10.3390/ijms161226171

**Published:** 2015-12-10

**Authors:** Sorina Nicoleta Petrache Voicu, Diana Dinu, Cornelia Sima, Anca Hermenean, Aurel Ardelean, Elena Codrici, Miruna Silvia Stan, Otilia Zărnescu, Anca Dinischiotu

**Affiliations:** 1 Department of Biochemistry and Molecular Biology, Faculty of Biology, University of Bucharest, 91–95 Splaiul Independentei, Bucharest 050095, Romania; sori.petrache@yahoo.com (S.N.P.V.); diana_dinu2006@yahoo.com (D.D.); miruna_stan@yahoo.com (M.S.S); otilia_zarnescu@yahoo.com (O.Z.); 2 Department of Experimental and Applied Biology, Institute of Life Sciences, Vasile Goldis Western University of Arad, 86 Rebreanu, Arad 310414, Romania; anca.hermenean@gmail.com (A.H.); biologie@uvvg.ro (A.A.); 3 Laser Department, National Institute of Laser, Plasma and Radiation Physics, 409 Atomistilor, Bucharest-Magurele 077125, Romania; cornelia.sima@inflpr.ro; 4 Department of Histology, Faculty of Medicine, Pharmacy and Dentistry, Vasile Goldis Western University of Arad, 1 Feleacului, Arad 310396, Romania; 5 Biochemistry Proteomics Department, Victor Babes National Institute of Pathology, 99-101 Splaiul Independentei, Bucharest 050096, Romania; raducan.elena@gmail.com

**Keywords:** SiO_2_ nanoparticles, heat shock proteins, oxidative stress, apoptosis, autophagy, MRC-5 cell line

## Abstract

This study evaluated the *in vitro* effects of 62.5 µg/mL silica nanoparticles (SiO_2_ NPs) on MRC-5 human lung fibroblast cells for 24, 48 and 72 h. The nanoparticles’ morphology, composition, and structure were investigated using high resolution transmission electron microscopy, selected area electron diffraction and X-ray diffraction. Our study showed a decreased cell viability and the induction of cellular oxidative stress as evidenced by an increased level of reactive oxygen species (ROS), carbonyl groups, and advanced oxidation protein products after 24, 48, and 72 h, as well as a decreased concentration of glutathione (GSH) and protein sulfhydryl groups. The protein expression of Hsp27, Hsp60, and Hsp90 decreased at all time intervals, while the level of protein Hsp70 remained unchanged during the exposure. Similarly, the expression of p53, MDM2 and Bcl-2 was significantly decreased for all time intervals, while the expression of Bax, a marker for apoptosis, was insignificantly downregulated. These results correlated with the increase of pro-caspase 3 expression. The role of autophagy in cellular response to SiO_2_ NPs was demonstrated by a fluorescence-labeled method and by an increased level of LC3-II/LC3-I ratio. Taken together, our data suggested that SiO_2_ NPs induced ROS-mediated autophagy in MRC-5 cells as a possible mechanism of cell survival.

## 1. Introduction

Silica (SiO_2_) represents one of the most common minerals on earth and a basic component of soil, sand, and rocks, including granite and quartzite. It can be found in both crystalline and amorphous forms. Silica nanoparticles (SiO_2_ NPs) are easy to prepare, inexpensive to produce and are used as additives or rheological modifier in the formulation of paints, plastics, and synthetic rubber. During the utilization of these materials, SiO_2_ NPs can be released in a time-dependent manner. Occupational exposure to crystalline silica dust is associated with a high risk for pulmonary diseases including silicosis, chronic bronchitis, and lung cancer [1,2], whereas amorphous silica is considered relatively safe.

Nanoparticles (NPs) are of great interest for a wide variety of potential applications in the fields of electronics, energy, and environmental and medical technology. The amorphous silica nanoparticles (SiO_2_ NPs) are among the most widely used nanomaterials, due to their advantages such as large surface area for loading biomacromolecules, biocompatibility, storage stability, and low cost production [3]. In the recent years, the use of silica nanoparticles for fundamental biomedical applications, including imaging [4], controllable drug delivery [5,6] and theranostics [7], as well as additives for food and cosmetics [8] has increased considerably.

Despite their widespread use, SiO_2_ NPs toxicity and safety to the human body and the environment have not been extensively investigated. To date, several *in vivo* and *in vitro* studies of the toxicity of SiO_2_ NPs have been performed. Previous *in vivo* studies suggested that the size of SiO_2_ NPs was critical for their toxicity. To be more specific, the treatment of mice with 70, 300, and 1000 nm SiO_2_ NPs revealed no hematological, histopathological or biochemical alterations in various organs suggesting these NPs can be employed in food production [9]; by contrast, exposure to 10–15 nm NPs resulted in toxic effects [10].

Similarly, cytotoxic effects induced by SiO_2_ NPs were reported in various cell lines such as HaCaT [11,12], H9c2(2-1) [13], Hek293 [14,15], EAHY926 [16], HepG2 [17,18], and A549 [19,20], with these effects being size- and dose-dependent, as well as highly cell type-dependent [21,22].

The mechanism by which SiO_2_ NPs induce toxicity is not clear. The formation of reactive oxygen species (ROS) can be considered as a possible mechanism for SiO_2_ NPs taking into consideration that crystalline silica has been shown to cause oxidative and inflammatory responses [23]. In addition, a significant increase in ROS production after SiO_2_ NPs exposure has been reported in several cell types [14,24,25,26].

It is generally accepted that increased quantities of ROS initiate lipid peroxidation in the cellular, mitochondrial, and nuclear membranes resulting in the degradation of cytosolic proteins and DNA damage [27]. So far, very few studies have investigated the effects of SiO_2_ NPs on cellular components. Lipid peroxidation occurrence [28], reduced glutathione (GSH) depletion [19], and DNA damage [25,29] have been previously reported, while the possible effects of SiO_2_ NPs on proteins have not been studied. Recently, SiO_2_ NPs were shown to induce ROS-mediated apoptosis in the human liver HepG2 cell line [30] and in the human lung epithelial A549 cell line [31].

Cell survival during stress requires the induction of the heat shock response. High levels of heat shock proteins (Hsps) can be triggered after exposure to various environmental stress conditions, such as increased temperature, presence of environmental pollutants and free radicals [32]. Thus, studying the relation between oxidative stress and cell death in SiO_2_ NPs exposure may be an important goal in order to elucidate the effects of these nanoparticles on cells.

Our study aimed to highlight the biochemical mechanisms responsible for the toxic effects of 7 nm size SiO_2_ NPs on a human lung fibroblast cell line (MRC-5). Mechanisms of ROS production and their effects on proteins, as well as the modulation of heat shock proteins’ expressions, were investigated. Apoptosis and autophagy, two processes by which damaged cells or organelles are eliminated, were also analyzed.

## 2. Results

### 2.1. Physico-Chemical Characterization of SiO_2_ Nanoparticles (NPs)

**Figure 1:**
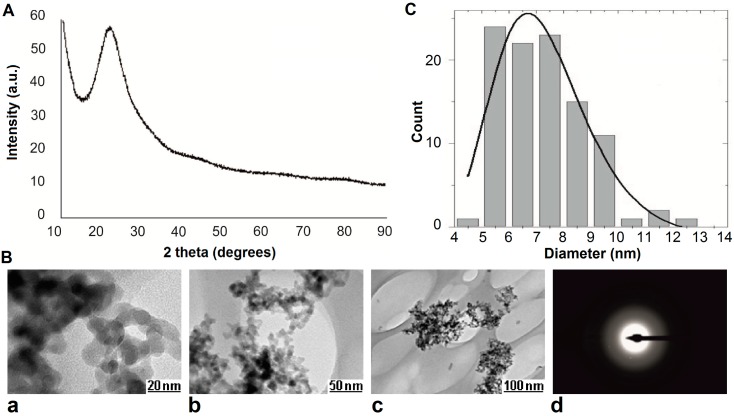
(**A**) XRD spectrum of SiO_2_ nanoparticles (NPs); (**B**) high resolution transmission electron microscopy (HRTEM) (**a**–**c**) and selected area electron diffraction (SAED) (**d**) images of SiO_2_ NPs; (**C**) Size distribution of SiO_2_ NPs.

The characterization of any type of NPs as far as their physicochemical properties are concerned is imperative for any nanotoxicological study [33]. The XRD spectrum (Figure 1A) showed that SiO_2_ NPs are amorphous, a result which was supported by the selected area electron diffraction (SAED) image (Figure 1B(d)). The spherical aspect of the SiO_2_ NPs can be observed in the high resolution transmission electron microscopy (HRTEM) images presented in Figure 1B(a–c). Also, it can be observed that the particles are agglomerated in clusters. Based on a statistics from electron microscopy images, the size distribution was obtained. The NP size distribution, which is a lognormal function, is shown in Figure 1C. The primary sizes of nanoparticles are between 4 and 13 nm with an average value of approximately 7 nm.

The hydrodynamic size of SiO_2_ NPs was measured in different suspensions (ultrapure water, cell culture media—Minimum Essential Medium (MEM) with/no fetal bovine serum). Compared to the primary size (7 nm) the nanoparticles showed a tendency to aggregate in aqueous media, reaching diameters of 156 ± 1.55 nm in ultrapure water; 157 ± 2.66 nm in culture medium MEM without fetal serum, and 159 ± 2.85 nm in culture medium MEM with 10% fetal serum. The effect of aggregation highlighted by Dynamic Light Scattering (DLS) measurements were confirmed by HRTEM images (Figure 1B(b,c)). Considering that the values obtained were almost equal, this suggests that there were no differences between the dispersion media used.

### 2.2. Cell Viability

**Figure 2:**
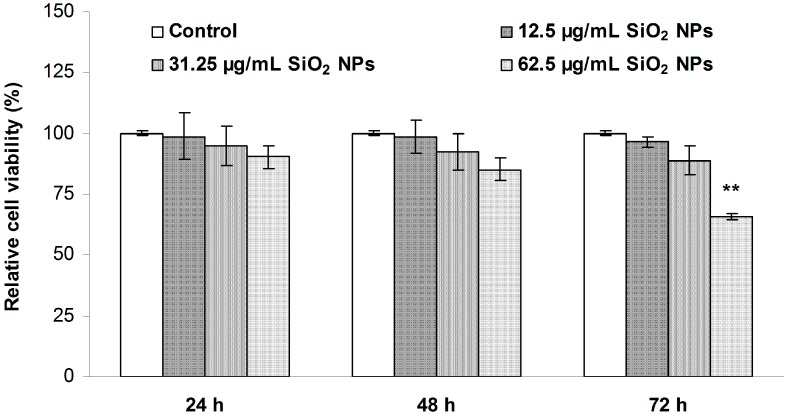
The viability of MRC-5 human lung fibroblast cells exposed to SiO_2_ NPs, at different concentrations, for 24, 48, and 72 h. Values are calculated as means ± SD (*n* = 3) and are expressed as % from controls. ** *p* < 0.01 *vs.* controls.

The cell viability after the exposure of MRC-5 fibroblasts to different concentrations of SiO_2_ NPs (12.5, 31.25 and 62.5 µg/mL) for 24, 48, and 72 h are presented in Figure 2. No significant changes were observed in the cells exposed to 12.5 and 31.25 µg/mL SiO_2_ NPs after 24, 48, and 72 h, respectively. Nevertheless, a 35% decrease in viability was observed when cells were exposed to the highest concentration of SiO_2_ NPs for 72 h, with no change being recorded for this dose after 24 and 48 h. This result indicates that the viability of MRC-5 cells decreased in a time- and dose-dependent manner. Taking into account these data, the final concentration of SiO_2_ NPs used in subsequent experiments was 62.5 µg/mL.

### 2.3. Morphological Changes Induced by SiO_2_ NPs in MRC-5 Human Lung Fibroblast Cells

**Figure 3:**
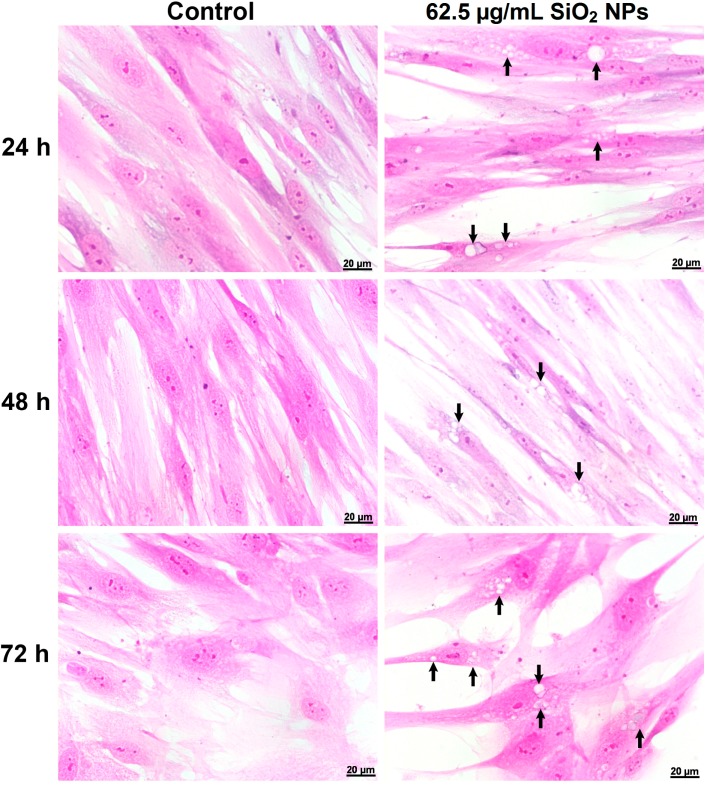
Cellular morphology of MRC-5 cells untreated (control) and exposed to 62.5 µg/mL SiO_2_ NPs for 24, 48 and 72 h. The arrows indicate the vacuolization of the cytoplasm during the exposure to SiO_2_ NPs. Scale bars = 20 μm.

A marked vacuolization of the cytoplasm was observed after the treatment of MRC-5 cells with 62.5 µg/mL SiO_2_ NPs (Figure 3). This vacuolization was not observed in the controls.

### 2.4. SiO_2_ NPs Induce Reactive Oxygen Species (ROS) Generation in MRC-5 Cells

**Figure 4:**
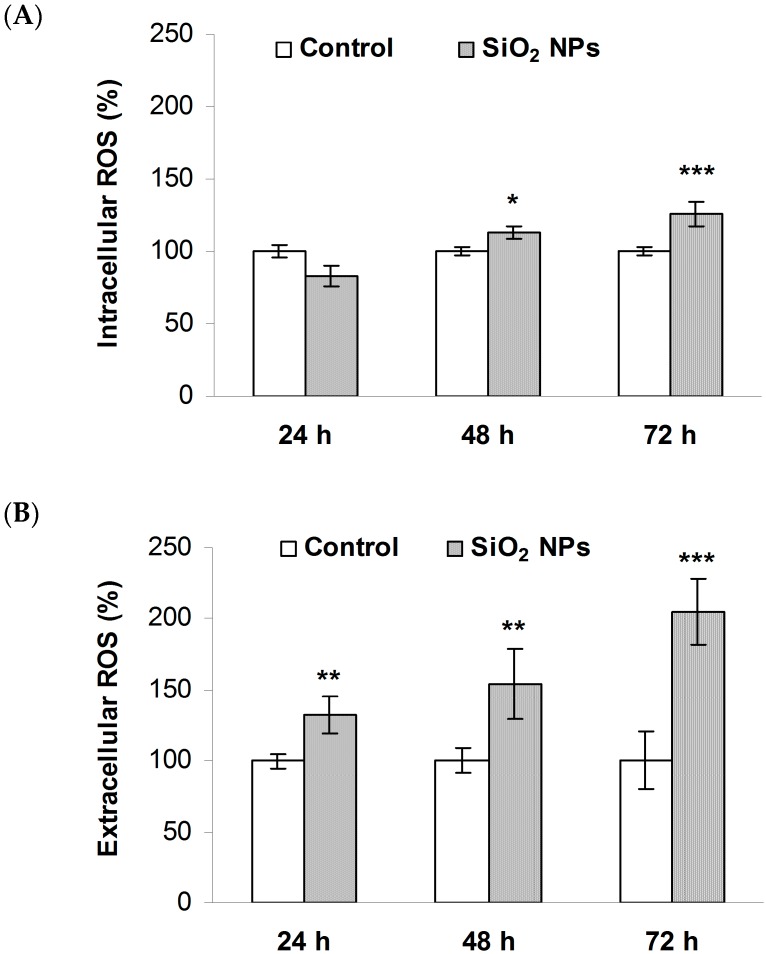
Reactive oxygen species (ROS) production in MRC-5 cells exposed to SiO_2_ NPs: (**A**) intracellular and (**B**) extracellular ROS concentrations. Values are calculated as means ± SD (*n* = 3) and are expressed as % from controls. * *p* < 0.05 *vs.* controls; ** *p* < 0.01 *vs.* controls; *** *p* < 0.001 *vs.* controls.

A significant increase of both intra- and extracellular ROS levels were observed in MRC-5 cells treated with 62.5 µg/mL SiO_2_ NPs. After 24 h of exposure, the fluorescence intensity of 2′,7′-dichlorodihydrofluorescein (DCF) was unchanged, but it became higher in SiO_2_ NPs treated cells, by 12% and 26% after 48 and 72 h, respectively, suggesting an increase in ROS generation (Figure 4A). The extracellular ROS concentration increased by 32%, 54%, and 104% after 24, 48, and 72 h, respectively (Figure 4B).

### 2.5. Glutathione (GSH) Concentration

**Figure 5:**
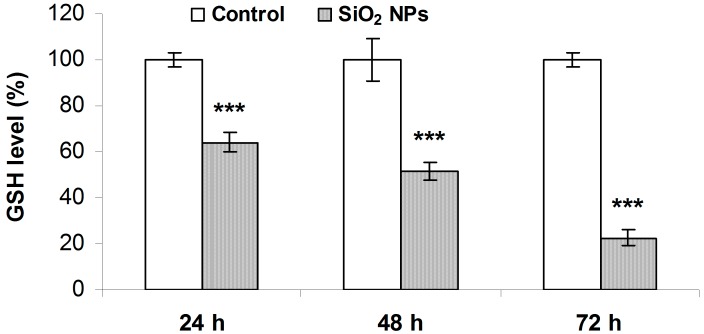
Glutathione (GSH) level in MRC-5 cells treated with SiO_2_ NPs. Values are calculated as means ± SD (*n* = 3) and are expressed as % from controls. *** *p* < 0.001 *vs.* controls.

A time-dependent decrease of the GSH level was observed in the MRC-5 cells treated with SiO_2_ NPs. In more detail, GSH was diminished by 36%, 50%, and 78% after 24, 48, and 72 h, respectively (Figure 5).

### 2.6. Protein Oxidative Modifications

The effects of the SiO_2_ NPs exposure on protein oxidation in MRC-5 cells are summarized in Table 1. In our experiment, advanced oxidation protein products (AOPP) levels were increased by 10%, 32% and 77% after 24, 48, and 72 h, respectively (Table 1). In addition, the protein thiol (PSH) levels were reduced by 20% and 35%, after 48 and 72 h, respectively, with no significant changes being recorded for the 24 h time point (Table 1). Furthermore, the protein carbonyl groups, another index of protein oxidative modification, was increased by 11% and significantly elevated by 51%, after 48 and 72 h of treatment with SiO_2_ NPs (Table 1).

**Table 1:** Advanced oxidation protein products (AOPP), protein carbonyl (PCG) and protein thiol (PSH) groups in MRC-5 cells after 24, 48 and 72 h of SiO_2_ nanoparticles (NPs) exposure. Results are means ± SD (*n* = 3) and are expressed as % from controls.

Time (h)	AOPP (nmoles/mg)		PSH (nmoles/mg)		PCG (nmoles/mg)	
	Control Cells	Exposed Cells	Control Cells	Exposed Cells	Control Cells	Exposed Cells
24	100 ± 2.27	109.39 ± 2.55	100 ± 1.79	98.4 ± 1.85	100 ± 3.78	97.18 ± 9.69
48	100 ± 2.44	131.66 ± 11.66 ***	100 ± 1.80	83.61 ± 4.33 ***	100 ± 2.54	111.18 ± 4.56 **
72	100 ± 2.13	176.47 ± 2.96 ***	100 ± 6.10	67.02 ± 4.42 ***	100 ± 5.54	150.98 ± 8.78 ***

** *p* < 0.01 *vs.* controls; *** *p* < 0.001 *vs.* controls.

### 2.7. Heat Shock Proteins Expression

**Figure 6:**
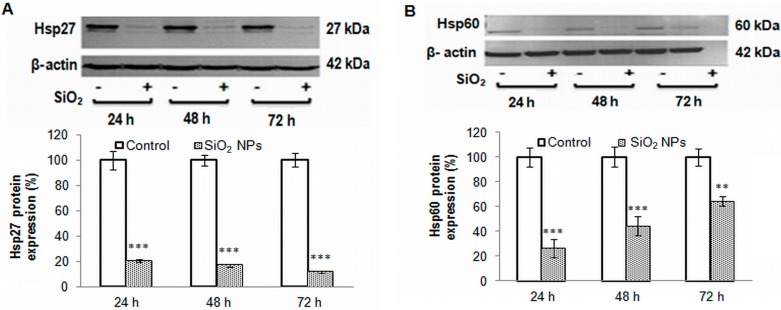

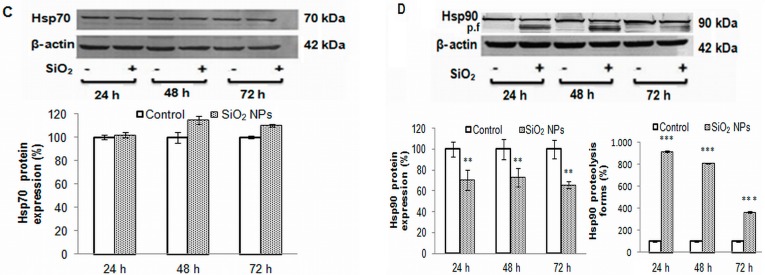
Representative Western blot images and quantitative analyses of Hsps expression in MRC-5 cells after the treatment with 62.5 µg/mL of SiO_2_ NPs for 24, 48, and 72 h. (**A**) Hsp27; (**B**) Hsp60; (**C**) Hsp70 and (**D**) Hsp90 and Hsp90 proteolysis form (p.f). The protein expression was normalized to β-actin. Results are expressed as means ± SD (*n* = 3) and are represented as % from controls. ** *p* < 0.01 and *** *p* < 0.001 *vs.* controls.

Western blot studies showed that Hsp27 expression was strongly inhibited in the SiO_2_ NPs treated cells, by 80%, 83%, and 89%, after 24, 48, and 72 h, respectively (Figure 6A). A significant decrease in Hsp60 expression, by approximately 74%, was registered in MRC-5 cells after 24 h of treatment. For longer time exposures, Hsp60 expression appeared to be restored as it increased by 56% and 36% after 48 and 72 h, respectively (Figure 6B). The levels of Hsp70 did not change in MRC-5 cells after SiO_2_ NPs exposure (Figure 6C). A down-regulation of 30% was registered for Hsp90 during the entire 72 h exposure to SiO_2_ NPs (Figure 6D). Furthermore, the appearance of an additional Hsp90 protein band in MRC-5 cells treated with NPs was noticed (Figure 6D).

### 2.8. The Main Proteins Involved in Apoptosis

**Figure 7:**
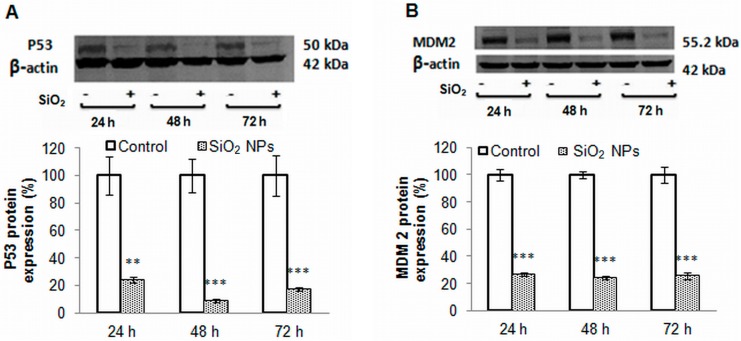

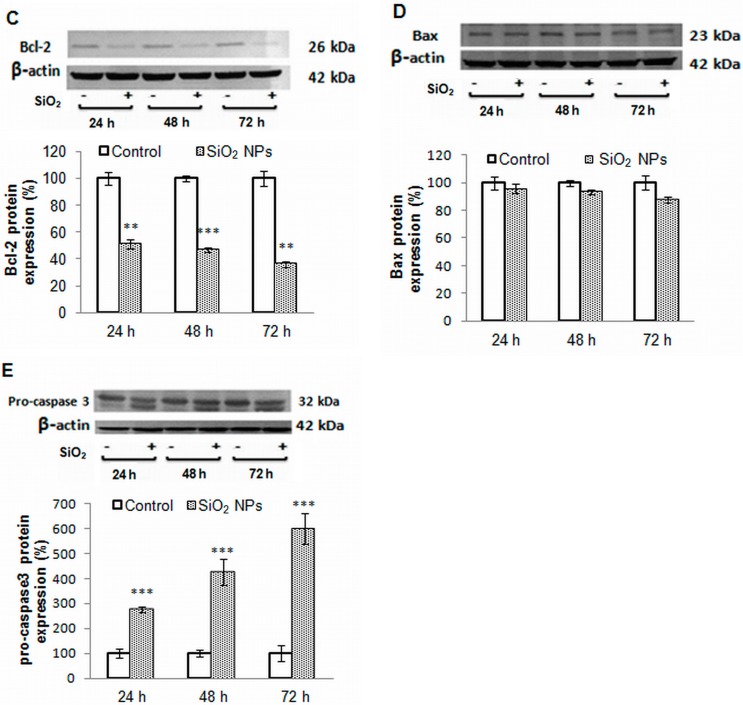
Representative Western blot images and quantitative analyses of p53 (**A**); MDM2 (**B**); Bcl-2 (**C**); Bax (**D**) and pro-caspase 3 (**E**) proteins expression in MRC-5 cells exposed to SiO_2_ NPs. The protein expression was normalized to β-actin. Results are expressed as means ± SD (*n* = 3) and are represented as % from controls. ** *p* < 0.01 and *** *p* < 0.001 *vs.* controls.

The protein expression level of p53, MDM2, Bcl-2, Bax and pro-caspase 3 were quantified by Western blot analysis. Our results showed that the level of p53, a tumor suppressor, decreased significantly after 24, 48, and 72 h of treatment by 76%, 91%, and 82%, respectively (Figure 7A). The expression of MDM2 was decreased by 75% (Figure 6B) for all time intervals. Also, Bcl-2 expression decreased in a time-dependent manner (Figure 7C), while Bax expression remained unchanged (Figure 7D). A time-dependent increase, by 176%, 328%, and 500% after 24, 48, and 72 h, respectively, was observed for procaspase-3 (Figure 7E).

### 2.9. Autophagy Induced by SiO_2_ NPs

**Figure 8:**
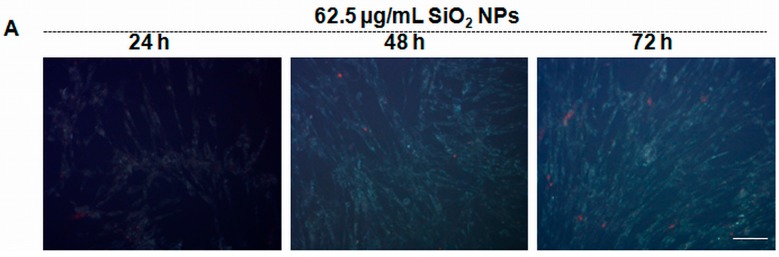

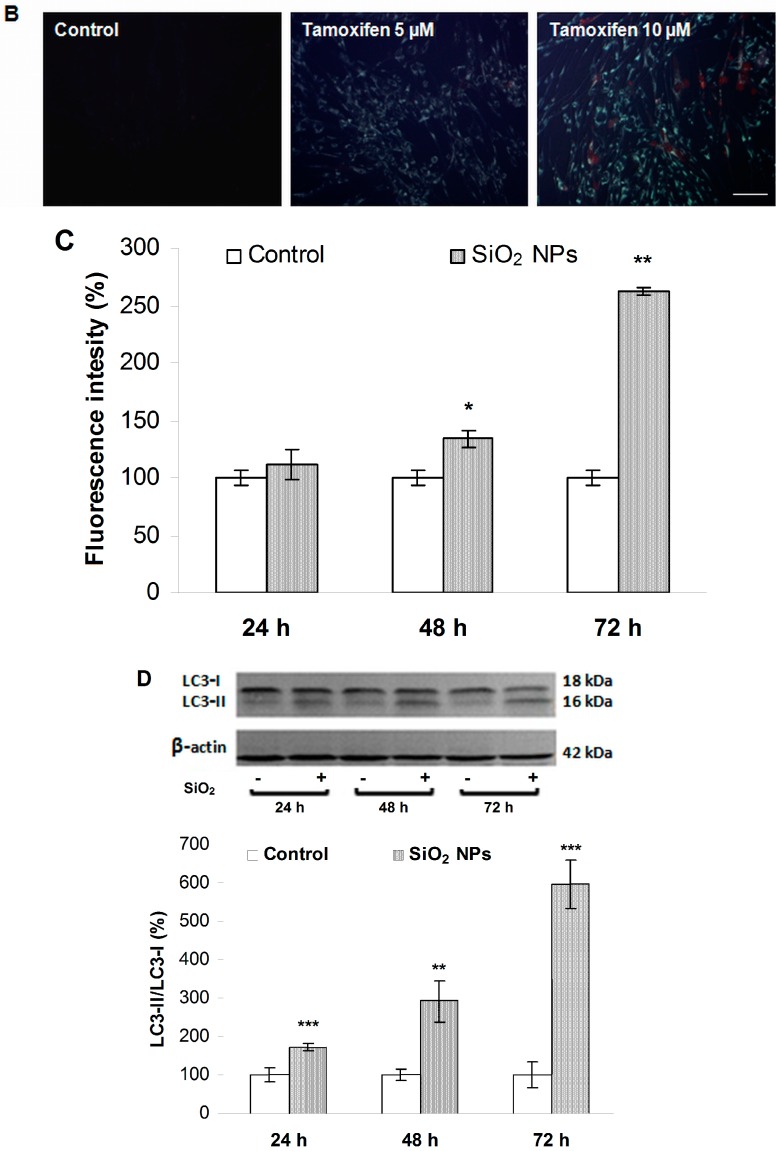
Autophagy induced in MRC-5 cells by SiO_2_ NPs. (**A**) MRC-5 cells exposed to 62.5 µg/mL silica nanoparticles for 24, 48, and 72 h were stained with monodansylcadaverine (MDC) to detect autophagic vacuoles and were counterstained with propidium iodide for cell death (Scale bar, 100 µm); (**B**) MRC-5 control cells and those exposed to 5 and 10 µM Tamoxifen, as a positive control of autophagy (Scale bar, 100 µm); (**C**) Quantification of MDC fluorescence intensity by Image J software (National Institutes of Health, Bethesda, MD, USA); (**D**) Representative Western blot images of LC3-I and LC3-II proteins and quantitative analyses of LC3-II/LC3-I ratio. The protein expression was normalized to β-actin. Results are expressed as means ± SD (*n* = 3) and are represented as % from controls. * *p* < 0.05; ** *p* < 0.01 and *** *p* < 0.001 *vs.* controls.

Figure 8A,C showed that the fluorescence intensity of monodansylcadaverine (MDC) staining after exposure to 62.5 µg/mL SiO_2_ NPs increased progressively by 11%, 34%, and 162% after 24, 48, and 72 h, respectively. In addition, the LC3-II/LC3-I ratio increased in a time dependent manner by 71%, 190%, and 497% after 24, 48, and 72 h, respectively, compared to control (Figure 8D).

## 3. Discussion

Given that SiO_2_ NPs have gained increasing interest for biomedical applications recently, it is important to evaluate their potential adverse effects. Taking into consideration that toxicity of SiO_2_ NPs is the main limitation of their applications, it is necessary to understand the biological response following their administration. Recently, a series of studies showed that small size SiO_2_ NPs are more harmful compared to the large ones [34,35,36,37]. Thus, our study investigated the biochemical effects induced by ultra-fine SiO_2_ NPs (7 nm diameter) in MRC-5 cells.

Oxidative stress is the most discussed paradigm for NPs mediated toxicity [38,39]. Its generation is a consequence of the imbalance between ROS formation and the response of the cellular antioxidant system. The SiO_2_ NPs in our study were also able to generate intracellular and extracellular ROS (Figure 3). Surface chemistry of silica NPs is governed by the concentration of hydroxyl groups and surface defects [40]. Recently, the ability of amorphous silica sub-micron powders to generate hydroxyl radicals was reported [41]. As a result, SiO_2_ NPs could produce ROS in the culture medium by chemical reactions and/or by activating membrane NADPH (nicotinamide adenine dinucleotide phosphate reduced) oxidases that catalyze the reaction which produces superoxide anion. The charge of the extracellular superoxide makes it unable to cross cellular membranes [42] but it can penetrate them through a chloride channel [43]. Also, taking into account that fibroblasts are an important source of extracellular superoxide dismutase (SOD) [44] this enzyme could catalyze the reaction of superoxide dismutation, generating hydrogen peroxide, which has been shown to be relatively stable and can easily diffuse within cells through aquaporins [45]. On the other hand, after their cellular uptake, NPs might interact with NADPH oxidases from the endoplasmic reticulum [46] and/or with mitochondria, disturbing the electron transport chain and generating superoxide anion from molecular oxygen via one-electron reduction and further reductions, ultimately leading to hydrogen peroxide formation.

The cellular defense pathways can regulate the level of ROS under normal metabolic conditions and protect against harmful oxidants. This defense system involves both antioxidant enzymes and free radical scavengers with low molecular weight, the most important one being the tripeptide glutathione (GSH) [47]. The mechanisms of antioxidant defense include various enzymes, such as superoxide dismutase (SOD), catalase (CAT), and glutathione peroxidase (GPX). SOD disintegrates superoxide radical by converting it to hydrogen peroxide, which is further destroyed by CAT and GPX. The up-regulation of these antioxidant enzymes, showed in our previous study [28], seems to be insufficient to counteract the ROS production by SiO_2_ NPs in MRC-5 cells.

Having an active thiol group as a cysteine residue, GSH plays an antioxidant role through a direct interaction with ROS and electrophiles or by acting as a cofactor for different enzymes such as GPX and glutathione-*S*-transferase (GST). GPX mainly catalyzes the direct reaction of GSH with ROS such as hydrogen peroxide and lipid peroxides, while GSTs exhibit GSH-dependent catalytic activities including the reduction of organic hydroperoxides.

The decline in GSH level in MRC-5 cells treated with SiO_2_ NPs, observed in our studies, was time-dependent (Figure 5). Previous studies reported decreases in GSH level in various cellular types after SiO_2_ NPs exposure [14,24,48]. In our preliminary investigations, GPX and GST activities were significantly increased in MRC-5 cells, after 48 and 72 h of SiO_2_ NPs exposure [28], with the last period of exposure being characterized by a severe decrease of the GSH level. These results indicate that GPX and GST may play a major role in SiO_2_ NPs induced GSH depletion. The role of these enzymes in the GSH decline as a consequence of silica nanoparticles toxicity been previously reported [48,49].

Nevertheless, the antioxidant system adaptation to SiO_2_ NPs was insufficient to prevent ROS formation and thus, biomolecule damage occurred.

Proteins, the most abundant and diverse molecules found in living cells, are important targets of ROS attack. The increase of advanced oxidation protein products (AOPP) [50] and protein carbonyls (PCG) formation [51], together with the decrease in protein-thiols (PSH) level [52], were reported as oxidative alterations of these biomolecules. These protein changes have not been sufficiently investigated in connection with SiO_2_ NPs exposure. In our experiments, an increase in AOPP level was noticed in MRC-5 cells (Table 1). Similar results were reported in *Carassius auratus gibelio* muscle [53], kidney [54], and liver [55], exposed to Si/SiO_2_ quantum dots. *In vitro* studies showed that AOPPs were able to inhibit inducible NO production by macrophages, to induce inflammation in fibroblast-like synoviocytes through a NADPH oxidase-dependent activation of NF-κB [56] and to up-regulate ROS concentration in human umbilical vein endothelial cells by activating NADPH oxidase, ERK 1/2, p38 and NF-κB [57]. Our results, showing a moderate decrease in PSH level (Table 1), were in agreement with those reported for human cerebral endothelial cells (HCEC) exposed to 25 nm silica nanoparticles [58]. ROS especially attack the thiol groups and sulphur-containing amino acids. A proton from cysteine residues can be abstracted by the activated oxygen, forming a thiyl radical that will cross-link to another thiyl radical to form intramolecular or intermolecular disulphide bridges. By reacting with ROS, thiol groups function as antioxidants. In this way, PSH are able to scavenge more than half of the intracellular generated ROS, causing reversible or irreversible oxidations to them during this step [59]. Carbonylation, an irreversible ROS-induced protein modification, showed a notable increase only at 72 h after SiO_2_ NPs treatment (Table 1). There are many ways to introduce carbonyl groups into a protein structure: ROS may directly oxidize lysine, arginine, proline, and threonine residues, or may react with carbohydrates and lipids generating reactive carbonyl species (RCS), such as ketoamine, ketoaldehydes, malondialdehyde, and 4-hydroxy-2-nonenal, which subsequently interact with proteins [51]. The physical and chemical properties of proteins, such as conformation, structure, solubility, and susceptibility to proteolysis, can be affected by the oxidative modification which may affect their physiologic functions. Thus, the increased levels of protein oxidation observed after SiO_2_ NPs exposure can be associated with various biological consequences in MRC-5 cells.

Hsps fulfill major functions in the folding, unfolding, and translocation of proteins, but also in the assembly and disassembly of protein complexes; they are essential for breaking-down of irreversibly denatured proteins resulting from oxidative stress [60] and have a major contribution to the maintenance of the cell homeostasis [61]. Given the particular role of these proteins, the expression of selected Hsps was investigated. To our knowledge, the modulation of these proteins’ expression (Hsp27, Hsp60, Hsp70, and Hsp90) in MRC-5 cells following SiO_2_ NPs treatment has not been studied to date. Hsp90 is involved in the formation of correct conformation and the activation of more than 200 client proteins in the eukaryotes [62]. One prominent Hsp90 client protein is the tumor suppressor protein p53 [63]. The recruitment of client proteins requires the collaboration of Hsp90 with Hsp70 and other co-chaperones [64]; subsequently, the resulting chaperone complex acts together with the ubiquitin-proteasome system for protein quality control [65]. ROS can provoke the cleavage of Hsp90, promoting the alteration of its chaperoning function due to removal of very important amino acid residues from the N-terminal domain that interact with nucleotides [66]. As a result, probably, p53 is not folded correctly and its expression is down-regulated significantly. At the same time, it is possible that hydroxyl radical generation in the presence of NADH (nicotinamide adenine dinucleotide reduced), ferrous iron, and hydrogen peroxide [67] decreases cellular NADH level and as a result, ubiquitin-independent p53 degradation occurs [68].

Taking into account that p53 activates the expression of MDM2 [69] and Bax [70] at the transcriptional level, the decrease of p53 expression could be correlated with the down-regulation of these two proteins. Furthermore, the decrease of Bax expression impairs the release of cytochrome C from the mitochondrial inner membrane, which in turn compromises the activation of procaspase 9 and procaspase 3.

Our data proved a down-regulation of Hsp60 in MRC-5 cells exposed to SiO_2_ NPs, which could be due to NO generation, as previously shown [71]. Our results are in contradiction with those of Radu *et al.* [72], which suggests that the chemical nature of NPs is critical for the biological response. In addition, the low level of Hsp60 could be correlated with the Bcl-2 level [73].

Western blot studies showed that Hsp27 expression was strongly inhibited in SiO_2_ NPs treated cells (Figure 5A). Hsp27 responds to oxidative stress by directly inhibiting components of the apoptotic pathways [74]. A previous study reported the involvement of autophagy–lysosomal protein degradation process in the regulation of the Hsp27 content [75]. Also, the inhibition of proteasome led to the activation of autophagy [76], with Hsp27 being associated with elevated proteasome activity [77]. In our opinion, the reduction in Hsp27 expression may be due to the activation of autophagy observed in MRC-5 cells as a result of SiO_2_ NPs exposure (Figure 7). Autophagy, initiated by p53 down-regulation [78], is up-regulated under stress conditions, such as extracellular and intracellular stress induced by the accumulation of damaged proteins [79]; it either mediates survival or cell death. Although considered an adaptive survival mechanism [80], autophagy changes into cell death when unfolded and damaged proteins overwhelm the degradative capacity of the proteasome [81]. In our experiment, autophagic vacuoles appeared starting with 48 h of exposure. At the same time point, Hsp70 expression slightly increased in an insignificant manner. Given that overexpression of Hsp70 inhibits autophagy [60], it could be that MRC-5 cells tried to counteract the onset of this mechanism. During autophagy, double membrane autophagosomes assemble in order to engulf intracellular components. The cytosolic microtubule-associated protein 1A/1B-light chain 3 (LC3-I) is conjugated with phosphatidylethanolamine during the process of autophagosomal membrane formation and subsequently generates LC3-II [82]. Figure 8 reveals an increased number of dead cells in the sample treated with tamoxifen as positive control compared to the sample exposed to SiO_2_ NPs. This fact could suggest that MRC-5 cells exposed for up to 72 h to 62.5 μg/mL SiO_2_ NPs activated autophagy as a survival mechanism, since the cell viability did not decrease so much (only by 35% of control for the highest concentration of nanoparticles after 72 h). This percent of cell death could indicate the mitochondrial damage by autophagy taking into account that 3-(4,5-dimethilthiazol-2-il)-2,5-dipheniltetrazolium bromide (MTT) staining involved the measurement of succinate dehydrogenases activity. In addition, the induction of autophagy was confirmed by the increased LC3-II/LC3-I proteins ratio (Figure 8d).

Previously, it was proved that the *in vivo* exposure to silica nanoparticles induced oxidative stress and pro-inflammatory responses in mice [24]. It is also known that generation of ROS and cytokines are regulated by a positive feedback mechanism. Although the *in vitro* results cannot exactly predict the *in vivo* effects of SiO_2_ NPs, our data represent valuable knowledge for future animal nanotoxicity studies.

## 4. Experimental Section

### 4.1. Characterization of SiO_2_ NPs

SiO_2_ NPs were purchased from NaBond Technologies Co., Ltd. (Shenzhen, China). Their morphology, structure, and size distribution was characterized using high resolution transmission electron microscopy (HRTEM), selected area electron diffraction (SAED) and X-ray diffraction (XRD) methods. The high resolution TEM studies were carried out on a Philips CM120 model electron microscope (FEI Company, Eindhoven, The Netherland). The TEM samples were obtained through dispersions in ethyl alcohol and were placed on carbon holey copper grids. The solvent evaporation was done at room temperature. XRD measurements have been performed using CuKα radiation (CuKα1 1.540598 Å and CuKα2 1.544426 Å) at room temperature on a D8 Advance Powder X-ray Diffractometer from Bruker AXS (Karlsruhe, Germany). The sample scanning was performed with an angular step of 0.02 degrees and a speed of 0.4 deg·min^−1^.

The hydrodynamic size of SiO_2_ NPs was assessed on a Malvern Zetasizer Nano-ZS instrument (Malvern Instruments, Malvern, UK). SiO_2_ NPs stock suspension with a concentration of 12.5 mg/mL in ultrapure water was sonicated for 45 min at room temperature and was diluted to 62.5 µg/mL NPs in water and cell culture medium (Minimum Essential Medium, MEM, Invitrogen, Grand Island, NY, USA) containing 10% fetal bovine serum (FBS; Gibco, Grand Island, NY, USA) or not. The measurements were performed in triplicate after an equilibration time of 30 s at 25 °C using the refractive index of 1.48 which corresponds to SiO_2_.

### 4.2. Cell Culture and Exposure to SiO_2_ NPs

The human fetal lung fibroblast cell line (MRC-5) was purchased from the American Type Culture Collection (ATCC; Rockville, MD, USA) and cells were cultured in Minimum Essential Medium (MEM) containing non-essential amino acids, Earle’s salts, l-glutamine, and supplemented with 10% fetal bovine serum (Gibco), 1% antibiotic-antimycotic solution (containing 100 U/mL penicilin, 100 µg/mL streptomycin and 0.25 µg amphotericin B; Sigma-Aldrich, St. Louis, MO, USA); cells were maintained in a humidified air atmosphere with 5% CO_2_ at 37 °C. These were seeded onto 75 cm^2^ culture flask at a density of 2.5 × 10^5^ cells/mL and were used between passages 10 and 20. Before exposure, the stock suspension of SiO_2_ NPs with a concentration of 12.5 mg/mL was sterilized, sonicated for 45 min in the ultrasonic bath, and further diluted in cell culture medium with 10% FBS. The cells were incubated for 24, 48, and 72 h with 12.5, 31.25 and 62.5 µg/mL SiO_2_ NPs for cell viability test and with 62.5 µg/mL SiO_2_ NPs for the rest of assays. Controls without SiO_2_ NPs were run for each time exposure.

### 4.3. Cell Viability Assay

The cell viability was determined using the 3-(4,5-dimethilthiazol-2-il)-2,5-dipheniltetrazolium bromide (MTT) reduction assay [83]. MRC-5 cells were exposed to 12.5, 31.25 and 62.5 µg/mL SiO_2_ NPs for 24, 48 and 72 h. After each exposure interval, the cell medium was replaced with PBS containing 1 mg/mL MTT and incubated at 37 °C for 2 h. Subsequently, the formazan crystals were solubilized in 2-propanol and the absorbance was read at 595 nm using a microplate reader (Tecan, GENious, Grödic, Germany). The absorbance of untreated cells was represented as 100% viability.

### 4.4. Cell Morphology

To analyze the morphology of MRC-5 cells, these were cultured for 24, 48, and 72 h in the presence of 62.5 µg/mL SiO_2_ NPs and then fixed in Bouin’s solution, stained with hematoxylin and eosin (H&E) and visualized by fluorescence microscopy.

### 4.5. Detection of Intracellular ROS

Detection of intracellular ROS was assessed with the fluorescent compound 5,6-carboxy-2′,7′-dichlorodihydrofluorescein-diacetate (carboxy-H_2_DCFDA) according to the instructions provided by the manufacturer (Invitrogen-Molecular Probes, Carlsbad, CA, USA). Carboxi-H_2_DCFDA compound enters the cell where it is hydrolyzed by intracellular esterases to form the highly fluorescent compound dichlorofluorescein (DCF).

MRC-5 cells were seeded in six-well plates at a density of 2 × 10^5^ cells per well and were allowed to attach for 24 h before treatment. After the cells were treated for 24, 48, and 72 h, the medium was changed to a Hank’s balanced salt solution (HBSS) buffer containing 25 μM of carboxy-DCFDA and incubated at 37 °C for 45 min. Subsequently, the cells were washed two times with HBSS buffer, treated with 0.25% trypsin and 0.03% EDTA, collected by centrifugation and resuspended in 1.5 mL of HBSS buffer. The fluorescence was quantified with a fluorimeter (Jasco, Tokyo, Japan) using an excitation wavelength of 495 nm and an emission wavelength of 529 nm. The results were expressed as % from control, after dividing the fluorescence intensity by the number of viable cells.

### 4.6. Extracellular ROS Assay

The amount of extracellular ROS in cells was assessed by chemiluminescence using luminol (5-amino-2,3-dihydro-1,4-phthalazinedione), according to the method described by Faulkner and Fridovich [84]. MRC-5 fibroblasts were seeded into 96-well cell culture plates at a density of 2 × 10^5^ cells per well in culture medium MEM with 10% fetal bovine serum. The cells were treated with 62.5 µg/mL SiO_2_ NPs at intervals of 24, 48, and 72 h. Volumes of 160 µL of the HBSS buffer and 40 µL of 6 mg% luminol in DMSO were added.

The chemiluminescence of control media and of SiO_2_ NPs treated samples was measured with an automatic Fluorimeter/Chemiluminometer system-Thermo Fluoroskan Ascent FL (Thermo Scientific, Rockford, IL, USA). The signals were registered for 50 min. Data processing was performed using the Microsoft Excel Software. Results were expressed as relative luminescence units (RLU). The measurements were performed three times in triplicate and the final results are expressed as % from control.

### 4.7. Reduced Glutathione Assay

The cellular lysates, deproteinized with 10% sulfosalicylic acid, were analyzed for total glutathione and oxidized glutathione (GSSG) using the Detect X^®^ Glutathione colorimetric detection kit (Arbor Assays, Ann Arbor, MI, USA) and following the manufacturer’s instructions. Reduced glutathione (GSH) concentration was obtained by subtracting the GSSG amount from the concentration of total glutathione. The GSH concentrations were assessed as nmoles/mg protein.

### 4.8. Protein Sulfhydryls Assay

The protein thiols (PSH) levels were measured by the modified method of Riener [85] based on the reaction between these thiol groups (-SH) and 4,4′-dithiodipyridine (DTDP) which leads to the formation of 4-pyridyldithio-derivative (RSS-Pyr). Protein samples were precipitated in the presence of 20% trichloroacetic acid (TCA) on ice for 10 min. The samples were centrifuged at 10,000 rpm at room temperature for 10 min. The precipitates obtained were solubilized in a minimum volume of 1 M NaOH, upon which 730 µL of 0.4 M Tris-HCl pH ~9 were added. Finally, a volume of 20 µL of 4 mM DTDP (4,4′-dithiodipyridine) prepared in 12 mM HCl was added and the absorbance of the mixture was measured at 324 nm. The concentration of protein sulfhydryl groups was measured using the *N*-acetyl cysteine standard curve and was expressed as nmoles/mg protein.

### 4.9. Advanced Oxidation Protein Products Assay

Spectrophotometric determination of the advanced oxidation protein products (AOPP) was carried out according to the method described by Witko-Sarsat [86] and modified by our group [54], using a chloramine-T standard curve. A volume of 200 µL cell lysate was homogenized with 10 μL of 1.16 M potassium iodide and vortexed for 5 min at room temperature. Subsequently, 20 μL of glacial acetic acid were added and the mixture was vortexed again for 30 s. The samples’ optical densities were read at 340 nm using a Tecan Genios Multireader. The AOPP level was expressed as nmoles/mg protein.

### 4.10. Protein Carbonyl Groups Assay

The concentration of protein carbonyl groups (PCG) was measured using the method of Levine [87] which was optimized in our laboratory [54]. A volume of 500 μL of cell lysate was mixed with 500 μL of 10 mM 2,4-dinitrophenylhydrazine prepared in 2 M HCl and incubated for 60 min at room temperature. After incubation, a volume of 500 μL of ice-cold 20% TCA was added and the mixture was kept on ice for 30 min. After centrifugation for 3 min at 13,000 rpm at room temperature, the supernatant was discarded and the pellet was washed twice with 1 mL (1:1) ethanol: ethyl acetate mixture. After 10 min of incubation at room temperature and another centrifugation, the pellet was suspended in 600 μL of 1 M NaOH and incubated for 15 min at 37 °C. The optical density of protein carbonyls was determined at 370 nm against a reagent blank and their concentration was calculated using the molar absorption coefficient of 22,000 M^−1^·cm^−1^. The results were expressed in nmoles/mg protein.

### 4.11. Western Blot Assays

The expression of heat shock proteins (Hsp27, Hsp60, Hsp70, and Hsp90), of the proteins involved in apoptosis (p53, MDM-2, Bax, Bcl-2, and pro-caspase 3) and of those implicated in autophagy (LC3) was analyzed by Western blot analysis. β-Actin was used as a reference protein. Western blot analyses were performed using cell lysates from harvested MRC-5 cells. A quantity of 30 µg of proteins was separated by 10% SDS-PAGE under reducing conditions at 90 V for 2 h in a TRIS-Glycine buffer. After electrophoresis, the separated proteins were transferred onto a 0.45 µm polyvinylidene difluoride (PVDF) membrane (Millipore, Billerica, MA, USA) at constant amperage (350 mA) for 120 min in a TRIS-glycine buffer.

The membranes were blocked for 60 min at room temperature using the blocking buffer included in the Western Breeze Chromogenic kit (Invitrogen, Grand Island, NY, USA). Subsequently, the membranes were incubated with appropriate dilutions of primary antibodies, overnight at 4 °C. The primary antibodies used were: anti-Hsp27, anti-Hsp60, anti-Hsp70, anti-Hsp90, anti-p53, anti-procaspase 3, anti-Bcl2, anti-Bax (Santa Cruz Biotechnology Inc., Santa Cruz, CA, USA), anti-MDM2 (Novus Biologicals, Colorado, CO, USA), anti-LC3 (NanoTools, Munich, Germany) and anti-β-actin (Sigma-Aldrich, St. Louis, MO, USA). Consecutively, the membranes were processed according to the instructions provided by the kit, using anti-mouse secondary antibodies coupled with alkaline phosphatase and 5-bromo-4-chloro-30-indolyphosphate/nitroblue tetrazolium (BCIP/NBT) as the chromogenic substrate. The Western Blot membranes were imaged using the GeneSys software (Syngene, Cambridge, UK) and the bands were quantified with the image processing and analysis application, Image J (National Institutes of Health, Bethesda, MD, USA). The relative values of the NPs-treated samples were expressed relative to the control of each experiment.

### 4.12. Autophagy Study

The autophagy induction in SiO_2_ NPs treated MRC-5 cells was established using the Autophagy/Cytotoxicity Dual Staining Kit (Cayman Chemical, Ann Arbor, MI, USA). This dual staining autophagy/cytoxicity cell-based assay facilitates the detection of both cells showing indicators of autophagy and cells showing indicators of cytotoxicity. The monodansylcadaverine (MDC) is an autofluorescent compound which can incorporate into multilamellar bodies through an ion trapping mechanism and upon the interaction with membrane lipids, and thus can serve as a probe in the *in vitro* detection of autophagic vacuoles. Propidium iodide staining was performed as a marker of cell death. Being a well-known inducer of autophagy, tamoxifen was used as a positive control in this assay.

MRC-5 cells, cultured at a density of 5 × 10^4^ cells per well in 96-well plates were treated with 62.5 µg/mL SiO_2_ NPs and different concentrations of tamoxifen (1–10 µM), for 24, 48, and 72 h. After the incubation period, the medium was removed, cells were washed with phosphate buffered saline (PBS) and then, a volume of 100 µL of appropriate diluted propidium iodide solution per well was added. After 10 min incubation at room temperature, the cells were washed with 100 µL wash buffer and treated for 10 min at 37 °C with 100 µL appropriate diluted solution MDC. Then the MDC solution was replaced with wash buffer and cells were immediately visualized by an Olympus IX71 fluorescence microscope (triple filter TRITC/FITC/DAPI, Olympus, Tokyo, Japan). The dead cells were revealed in red, while the autophagic vacuoles were shown in silver. The fluorescence was quantified using the image processing and analysis application, Image J.

### 4.13. Protein Concentration

The protein concentration was measured according to the method of Bradford [88] using bovine serum albumin as standard and was expressed as mg/mL.

### 4.14. Statistical Analysis

The results were expressed as mean values of triplicate experiments ± standard deviation (SD). The control and nanoparticles treated groups were compared by Student’s *t*-test using standard statistical packages. The data were considered significant for a *p* value less than 0.05.

## 5. Conclusions

In conclusion, our results show that exposure of lung fibroblasts to SiO_2_ NPs up to 72 h induced ROS generation, a decrease of GSH level, a raise of protein oxidation and at the same time, a down-regulation of Hsp27, Hsp60, and Hsp90 expressions as well as of p53 and MDM2 protein expression. Due to these biochemical events, apoptosis was inhibited and autophagy was promoted instead, possibly as a survival mechanism. Nevertheless, a longer treatment would probably direct autophagy towards cell death due to the low level of Hsp27, Hsp60, Hsp90 and GSH. However, SiO_2_ NPs of various sizes could exert different effects on MRC-5 cell line, as well as SiO_2_ NPs of same size might generate other consequences on other types of cells, so these issues should be further investigated.
